# Flagellar central pair assembly in *Chlamydomonas reinhardtii*

**DOI:** 10.1186/2046-2530-2-15

**Published:** 2013-11-27

**Authors:** Karl-Ferdinand Lechtreck, Travis J Gould, George B Witman

**Affiliations:** 1 Department of Cellular Biology, University of Georgia, 1000 Cedar Ave, 635 Biological Science Bldg., 30602 Athens, GA, USA; 2 Department of Cell and Developmental Biology, University of Massachusetts Medical School, Worcester, MA 01655 USA; 3 Department of Physics & Astronomy, Bates College, Lewiston, ME 04240, USA

**Keywords:** Hydin, Intraflagellar transport, Katanin, Microtubule

## Abstract

**Background:**

Most motile cilia and flagella have nine outer doublet and two central pair (CP) microtubules. Outer doublet microtubules are continuous with the triplet microtubules of the basal body, are templated by the basal body microtubules, and grow by addition of new subunits to their distal (“plus”) ends. In contrast, CP microtubules are not continuous with basal body microtubules, raising the question of how these microtubules are assembled and how their polarity is established.

**Methods:**

CP assembly in *Chlamydomonas reinhardtii* was analyzed by electron microscopy and wide-field and super-resolution immunofluorescence microscopy. To analyze CP assembly independently from flagellar assembly, the CP-deficient katanin mutants *pf15* or *pf19* were mated to wild-type cells. HA-tagged tubulin and the CP-specific protein hydin were used as markers to analyze *de novo* CP assembly inside the formerly mutant flagella.

**Results:**

In regenerating flagella, the CP and its projections assemble near the transition zone soon after the onset of outer doublet elongation. During *de novo* CP assembly in full-length flagella, the nascent CP was first apparent in a subdistal region of the flagellum. The developing CP replaces a fibrous core that fills the axonemal lumen of CP-deficient flagella. The fibrous core contains proteins normally associated with the C1 CP microtubule and proteins involved in intraflagellar transport (IFT). In flagella of the radial spoke-deficient mutant *pf14*, two pairs of CPs are frequently present with identical correct polarities.

**Conclusions:**

The temporal separation of flagellar and CP assembly in dikaryons formed by mating CP-deficient gametes to wild-type gametes revealed that the formation of the CP does not require proximity to the basal body or transition zone, or to the flagellar tip. The observations on *pf14* provide further support that the CP self-assembles without a template and eliminate the possibility that CP polarity is established by interaction with axonemal radial spokes. Polarity of the developing CP may be determined by the proximal-to-distal gradient of precursor molecules. IFT proteins accumulate in flagella of CP mutants; the abnormal distribution of IFT proteins may explain why these flagella are often shorter than normal.

## Background

Flagellar and ciliary motility function in cell locomotion and the generation of fluid flow. The majority of motile cilia contain a central pair apparatus (CP), which is comprised of two microtubules (C1 and C2) and associated projections and is involved in the regulation of dynein arm activity [1,2]. CP defects result in ciliary paralysis or abnormal ciliary motility and, at the organismal level, in infertility, hydrocephalus, and severe respiratory problems [3-7]. Numerous components of the CP have been identified [8,9], but our knowledge of how the CP is assembled is still very limited.

In contrast to the outer doublet microtubules of the axonemes, which are continuous with and templated by the A- and B-tubules of the basal body triplets, the CP microtubules are not anchored to the basal body. In *C. reinhardtii* and in ciliates the CP is twisted and probably rotates within the axonemal cylinder during flagellar beating, whereas in metazoans and *Trypanosoma* the CP has a fixed position and is often stably connected to the outer doublets by specialized radial spokes [10]; it is unclear whether these differences require fundamentally distinct pathways of CP assembly. Various mutations in the acidic C-terminal domains of tubulins result in the loss of the CP microtubules in *Tetrahymena* cilia and *Drosophila* sperm flagella suggesting that the assembly of the CP microtubules is particularly sensitive to tubulin quality [11-13]. The minus-end of the CP is positioned above the flagellar transition zone (TZ) [14], a structurally specialized region between the basal body and the axoneme proper. In *C. reinhardtii, γ*-tubulin has been localized to the TZ [15] and, in *Trypanosoma brucei*, *γ*-tubulin knock-down results in the loss of the CP from newly assembled flagella [16], suggesting that *γ*-tubulin near the transition region is involved in CP assembly. However, in *C. reinhardtii* cells with mutated centrin, the stellate structure that forms the central portion of the TZ is lost or partially lost without affecting CP assembly [17,18]. In these centrin mutants, CP microtubules were observed inside the basal body, suggesting that the stellate structure (or its equivalent in other organisms) functions as a barrier preventing the CP from sliding or growing into the basal body rather than being a CP nucleator. During *Drosophila melanogaster* spermatogenesis, a bona fide TZ is absent and a singlet microtubule, which constitutes the precursor of the CP, reaches from the short flagellum into the lumen of the basal body; it has been suggested that in this case the basal body functions as an organizing center for the CP [19]. Thus, the questions of how and where CP assembly is initiated are still unclear.

Here, we analyzed CP assembly during flagellar regeneration and repair in *C. reinhardtii* using the CP-specific protein hydin as a marker [20]. The assembly of the CP microtubules and its projections commences soon after the onset of outer doublet elongation. In the *C. reinhardtii* mutants *pf15* and *pf19*, defective in the regulatory and catalytic subunit of the microtubule-severing protein katanin, respectively [21,22], the CP is missing and replaced by an amorphous fibrous core [23,24]. After mating *pf15* or *pf19* to wild type (*WT*), motility is restored to the mutant-derived flagella by cytoplasmic complementation [25]. We used this approach to study CP assembly in full-length flagella and observed that the new CP initially became apparent in the subdistal region of the mutant-derived flagella. We conclude that CP assembly does not depend on proximity to the basal body or TZ or to the flagellar tip; rather, the CP appears to be able to self-assemble, with correct orientation, without the need for an organizing center. We also used biochemistry and super-resolution microscopy to determine that the electron-dense core which replaces the CP in the lumens of CP-deficient flagella contains IFT proteins and subunits of the C1 CP microtubule.

## Methods

### Strains and culture conditions

*C. reinhardtii* strains used in the work include 137c *(agg1*, *nit1*, *nit2*, *mt*+), CC124 *(agg1*, *nit1*, *nit2*, *mt*−), *pf6* (CC-929 mt-), and *pf6-2* (CC-3926 mt+), all of which are available from the *Chlamydomonas* Genetics Center; strain g1 (*nit1*, *agg1*, *mt+*) is described in Pazour et al. [26]. *pf15a* was obtained from R.P. Levine (Harvard University, Cambridge, MA, USA), whereas *pf18* and *pf19* were R. Lewin isolates originally obtained from the Culture Collection of Algae and Protozoa (Cambridge, UK); all have been maintained in the Witman laboratory since 1974. Cells were grown in M medium I [27] supplemented with 2.2 mM KH_2_PO_4_ and 1.71 mM K_2_HPO_4_ at 23°C with aeration and a light/dark cycle of 14/10 h. For gametogenesis, cells were spread onto TAP plates, grown for 6–8 days, and then transferred to dim light for 2–4 days. On the evening before the experiment, cells were resuspended in 6–10 mL of M-N (nitrogen-free M medium I) and incubated in constant light with agitation. In the morning, cells were transferred to diluted M-N medium (20% M-N, 10 mM Hepes) and incubated for 5 h in constant light.

### Immunofluorescence and electron microscopy

For immunofluorescence microscopy of zygotes, gametes were mixed and incubated for up to 70 min. Typically, samples were processed for immunofluorescence at various time points, e.g., 10, 20, 40, and 60 min after mixing of the gametes; incubation times varied slightly between experiments. Mating mixtures were transferred to HMEK (30 mM Hepes, 5 mM MgSO_4_, 5 mM EGTA, 25 mM KCl, pH 7) by centrifugation (3 min at 2,000 x *g*, room temperature), mixed with an equal volume of HMEK/3% Triton X-100/5–6% formaldehyde and applied to poly-L-lysine-coated (0.1% in water, Sigma) multi-well slides (Erie, Thermo Scientific) for 8–12 min depending on cell density. The slides were then plunged into −20°C methanol for 3–8 min and air-dried.

For standard immunofluorescence staining, vegetative cells in HMEK were mixed with an equal volume of either HMEK/0.5% Nonidet P-40/6% formaldehyde (simultaneous permeabilization and fixation) or HMEK/0.5% Nonidet P-40 (sequential permeabilization and fixation). In the latter case, the cell suspension was mixed 1:1 with 6% formaldehyde in HMEK soon (5–30 seconds) after cell lysis. The cell suspension was applied to polyethyleneimine-coated (0.1% in water) multi-well slides and allowed to settle for ~10–15 min. The slides were then washed in PBS, air-dried, blocked, and immunostained.

The following antibodies were used: anti-hydin (1:100; [20]), anti-hemagglutinin (HA) (1:200–800; Boehringer), anti-alpha-tubulin (1:800–1,200; Sigma), anti-acetylated tubulin (1:800; 6-11B-1; Sigma), anti-PF6 (1:300; [28]), anti-IFT139 and anti-IFT172 (each applied 1:1; [29]), and anti-IFT20 (1:100; [30]). After blocking (PBS/1% BSA/0.05% Tween20 for >30 min), wells were covered with primary antibody solution overnight, washed by submerging the slides in PBS, and incubated for 90–120 min with secondary antibody solution (Alexa Fluor 488, 568, or 594 conjugated to anti-rabbit, anti-mouse, or anti-rat antibodies diluted in blocking buffer). After several final washes with PBS/0.05% Tween 20, slides were submerged in ethanol for 10 seconds and dried. Mounting solution (Prolong Gold, Invitrogen) was applied to the wells, and the specimen was closed using a No. 1 cover glass. For triple immunofluorescence, specimens were first stained with rat anti-HA (Boehringer) and the rabbit primary antibody overnight. Then, anti-rat-488 was applied for ~60 min followed by incubation with the mouse-derived anti-tubulin antibodies and, subsequently, a mixture of anti-mouse-350 and anti-rabbit-594 secondary antibodies; sequential staining was necessary to avoid cross-reactivity of the anti-mouse secondary antibodies with the anti-rat-HA antibody.

Images were acquired at room temperature using AxioVision software and a camera (AxioCam MRm) on a microscope (Axioskop 2 Plus) equipped with a 100×/1.4 oil differential interference contrast Plan-Apochromat objective (Carl Zeiss Microimaging, Inc.) and epifluorescence. Image brightness and contrast were adjusted using Photoshop 6.0 (Adobe). Figures for publication were assembled using Illustrator 8.0 (Adobe). Capture times and adjustments were similar for images mounted together.

For stimulated emission depletion (STED) microscopy, *WT* and *pf19* cells were simultaneously and sequentially permeabilized and fixed as described above, allowed to settle on No. 1.5 cover glasses, washed with PBS, and incubated in blocking buffer. Air drying of specimens was omitted during the entire staining protocol to better preserve flagellar structure. Primary antibodies (anti-IFT172 and anti-β-tubulin) were applied overnight in blocking buffer at 4°C. Secondary antibodies (ATTO 647 N-goat anti-mouse IgG and Alexa Fluor 488 anti-rabbit IgG) were diluted 1:1,000 and applied for 1 h at room temperature. Specimens were mounted in 97% thiodiethylene glycol (Fluka Cat. No. 88559) supplemented with Prolong Gold antifade solution (Invitrogen). Specimens were analyzed using a two-color Leica TCS STED microscope.

For electron microscopy, cells were fixed in glutaraldehyde [31] and processed as described previously [32]. Time points during regeneration experiments were measured from the addition of acetic acid during pH shock. For analysis of *pf14* flagella, steady-state flagella were isolated, extracted with 0.5% Nonidet P-40, and centrifuged in a microcentrifuge tube. The pellet was then fixed and processed as previously described [32]. Specimens were examined using Philips CM10 or CM12 electron microscopes.

### Western blot analysis and isolation and fractionation of flagella

Flagella were isolated as previously described [33] and extracted with 1% Nonidet P-40 for 20–30 min on ice. After centrifugation (27,000 × *g*, 15 min, 4°C), the soluble phase (membrane + matrix) and the insoluble phase (axonemes) were collected and analyzed by SDS-PAGE and Western blotting using standard protocols. The following antibodies were used for Western blotting: anti-hydin (1:1,000), anti-PF6 (1:3,000), anti-CPC1 (1: 1,000; [34]), anti-KLP1 (1:1,000; [35]), anti-FAP114 (1:3,000; [28]), anti-IFT139 (1:100), anti-IFT172 (1:50), anti-IFT57 (1:50), anti-IFT81 (1:250), anti-DHC1b (1:1,000), anti-d1bLIC (1:800; [36]), anti-KAP (1:1,000), anti-BBS4 (1:1,000; [30]), and anti-IC2 (1:100; [37]).

## Results

### Ultrastructural analysis of CP assembly in regenerating flagella

**Figure 1:**
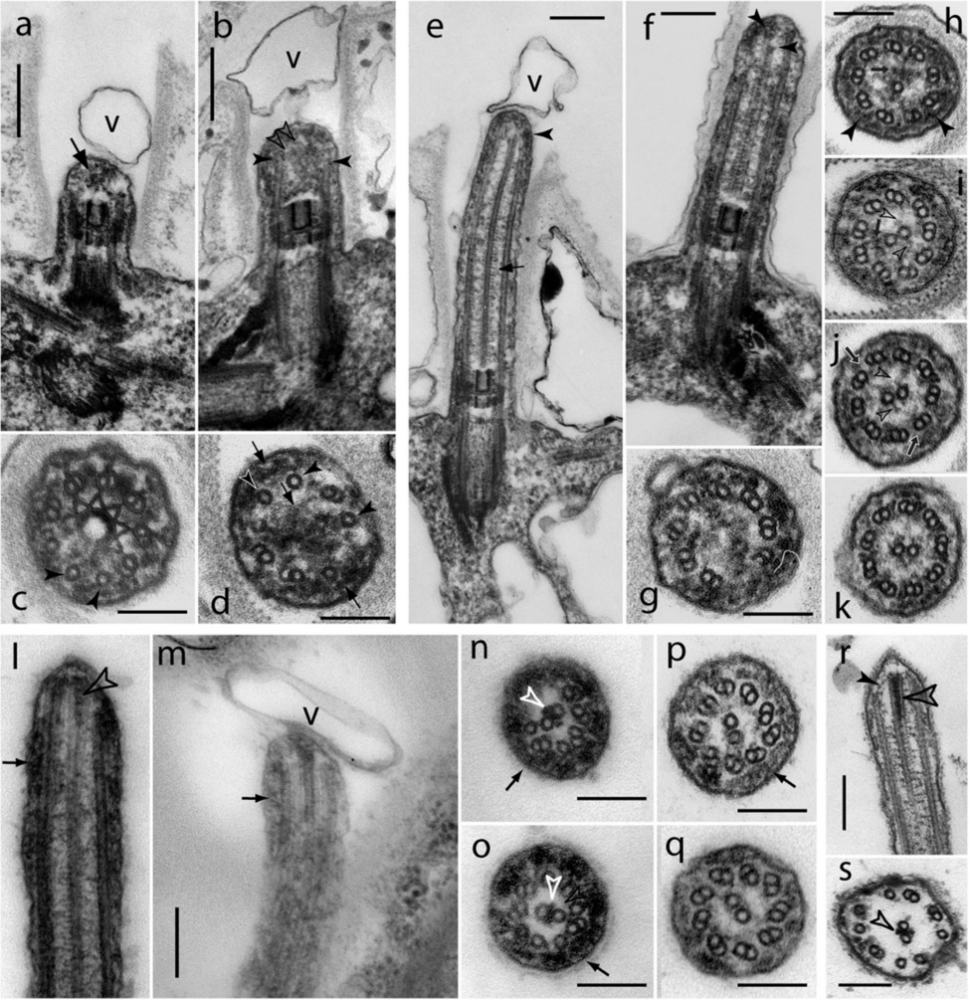
**Ultrastructure of short regenerating flagella.** Electron micrographs of cells fixed at various times after deflagellation (**a-q**). **r**, **s**: non-deflagellated control cells. **a-d**: very short regenerating flagella fixed at 7 minutes after deflagellation lack a bona fide CP. Arrows in a and **d**: granular material. Closed arrowheads in b: elongated microtubules. Open arrowheads in **b**: linear structures in the axonemal lumen which could represent a nascent CP. Arrowheads in c and **d**: singlet microtubules indicative of outer doublet formation. **e-k**: regenerating flagella at 14 minutes after amputation. Arrow in **e**: CP with projections. Arrowheads in **e** and **h**: fibrous material underlying the flagellar membrane. Arrowheads in **f**: staggered ends of the two CP microtubules. **g**, **h**: distal end of flagellum showing a ring of doublets without CP (**g**) and with a single CP microtubule (**h**). **i**: outer dynein arms are missing from the doublet microtubules but projections (open arrowheads) are visible on the CP. Small arrow in **h** and **i**: residual granular material in the axonemal lumen. **j**: projections are present on both CP microtubules (open arrowheads) but some outer dynein arms are missing (arrows). **k**: section revealing a full complement of dynein arms and CP projections. **l-s**: distal portions of regenerating flagella at 22 minutes after deflagellation (**l–q**) and of steady-state (**r**, **s**) flagella. Open arrowheads in **l**, **n**, **o**, **r**, and **s**: electron opaque tip sheet between the two CP microtubules. Arrows in **l**, **m**, **n**, **p** and **o**: fibrous material between the doublets and the membrane. Solid arrowhead in **r**: A-tubule cap forming a connection to the CP. V (in **a**, **b**, **e**, and **m**): vesicle at the flagellar tip. Bars = 200 nm (**a**, **b**, **e**, **f**, **l**, **m**, **r**) or 100 nm.

**Figure 2:**
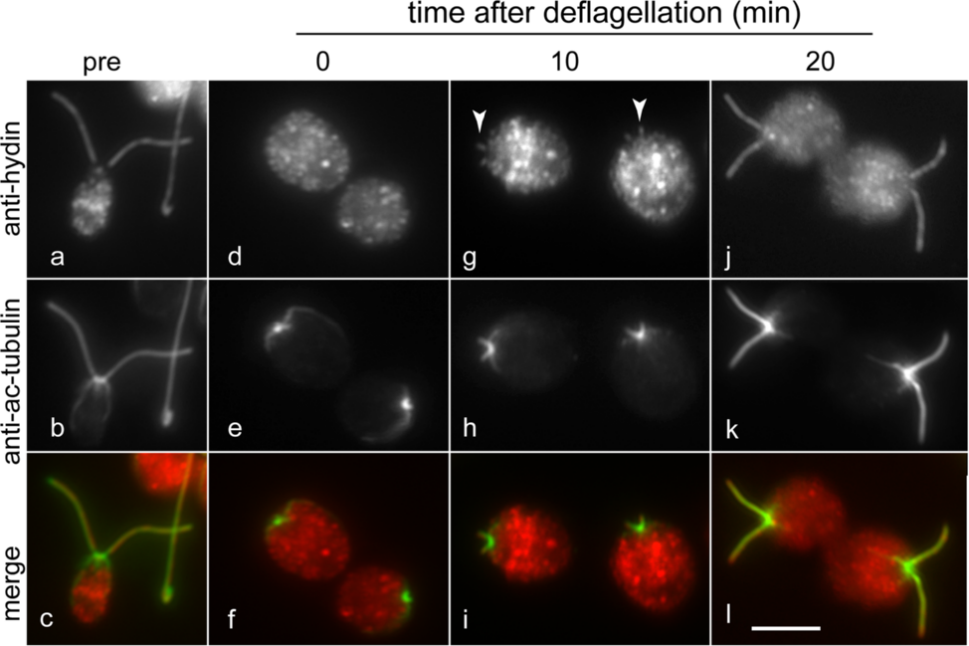
**Hydin is incorporated early during CP assembly.***WT* cells before (pre) and at various time points (0, 10, 20 min) after deflagellation were analyzed by immunofluorescence microscopy using anti-hydin **(a, d, g, j)** and anti-acetylated tubulin **(b, e, h, k)**. Merged images are shown in **c**, **f**, **i**, and **l**. Arrowheads in **g**: short flagella containing hydin. Bar = 5 μm.

To determine the timing of the formation of the CP microtubules and their projections during flagellar assembly, we performed thin-section transmission electron microscopy (TEM) of *C. reinhardtii* cells regenerating their flagella (Figure 1). Cells were fixed and embedded at 7 (T7), 14 (T14), and 21 min (T21) after deflagellation by pH shock. Longitudinal and cross sections of flagella at 7 min after deflagellation revealed singlet A-tubules indicative of the onset of outer doublet formation (Figure 1b,d). As previously noted [38], the nascent flagella contained a large amount of granular electron-opaque material that appeared to include IFT particles. CP microtubules were not detected in more than 15 cross-sections of T7 flagella analyzed. Two of five longitudinal sections, however, showed two elongated structures positioned diagonally relative to the outer doublets (Figure 2b, open arrowheads). This structure is also visible in Figure 22 of Rosenbaum et al. [38]; the increased density at the edges suggests that these structures might be tubular but their relationship to the CP is unclear. Regenerating flagella commonly have a vesicle attached to their tips (Figure 1a,b,e,m). In the sample fixed 14 min after deflagellation, when flagella were ~1 to 2 μm in length, a CP was visible in all appropriate sections (Figure 1e,f,i–k). Residues of the granular material that filled the axonemal lumen prior to CP assembly were still present (Figure 1h,i). The CP microtubules originated up to ~25 nm above the upper border of the H-like structure of the TZ [39]. Generally, the tip of the CP microtubules did not extend to the tip of the flagellum at this stage but was slightly shorter than the surrounding outer doublets (Figure 1 e,f,g,h). One longitudinal section showed that one of the two CP microtubules was distally ~100 nm longer than the other. CP projections were visible in axonemal cross-sections that lacked several outer dynein arms, suggesting that the assembly of projections onto CP microtubules preceded the assembly of a complete set of outer dynein arms at the same level (Figure 1i,j). In the T22 flagella, the CP exceeded the outer doublets in length as it does in steady-state flagella. Electron-opaque material, termed the “tip sheet” by Ringo [39], was present between the distal ends of the two CP microtubules at T22 (Figure 1l,n,o,r,s); this structure is characteristic of steady-state CPs but absent during early CP development. In T22 flagella, the tip region of growing flagella tapered and the tip of the axoneme was embedded in dense granular material. Radial spokes were absent from this region and the doublets were rotated inward so as to form a paddlewheel-like structure; frequently, one or two doublets were displaced inward so as to be in close proximity to the CP (Figure 1n–q). The data suggest two distinct phases of CP development: an initial phase during which the CP is fully enclosed in the axonemal cylinder and lacks the electron-opaque tip sheet, and a later phase when the CP projects from the axonemal cylinder and contains the characteristic tip sheet. CP assembly is slightly delayed relative to the formation of the doublet microtubules, and CP projections are added early.

### The CP protein hydin is present in short regenerating flagella

The 540-kD protein hydin is a component specific to the C2b projection, which is associated with the C2 microtubule of the CP [20]. To test when hydin is incorporated into the developing CP, cells were deflagellated by pH shock, fixed at various time points during flagellar regeneration, and double stained with anti-acetylated tubulin, a marker for flagellar tubulin, and anti-hydin (Figure 2). Hydin was readily detectable in short regenerating flagella (Figure 2g–i). Thus, the incorporation of hydin into the assembling CP occurs early during flagellar regeneration, in agreement with our ultrastructural observation that the CP projections are added early during CP development. Because hydin is almost completely absent from flagella of CP-deficient mutants [20], it can be used as a marker to determine the presence of the CP and study its assembly.

### Hydin accumulates in the middle segment of the flagellum during CP repair

During flagellar regeneration, the CP assembles while the flagellum is still very short. It is therefore unclear whether the CP assembles near the TZ because of these spatial constraints or because the TZ and neighboring flagellar region provide a unique environment for CP assembly. To analyze CP assembly independently of flagellar formation, we took advantage of the CP-deficient *C. reinhardtii* mutants *pf15* and *pf19*. These mutants have paralyzed flagella in which the CPs are replaced by amorphous electron-dense cores [23,24]. When gametes of these mutants are mated to *WT* gametes, motility is quickly restored to the formerly paralyzed flagella in the resulting zygotes [25], indicating that new CPs have been formed. This system thus provides an opportunity to analyze CP assembly independently from that of the outer doublets.

**Figure 3:**
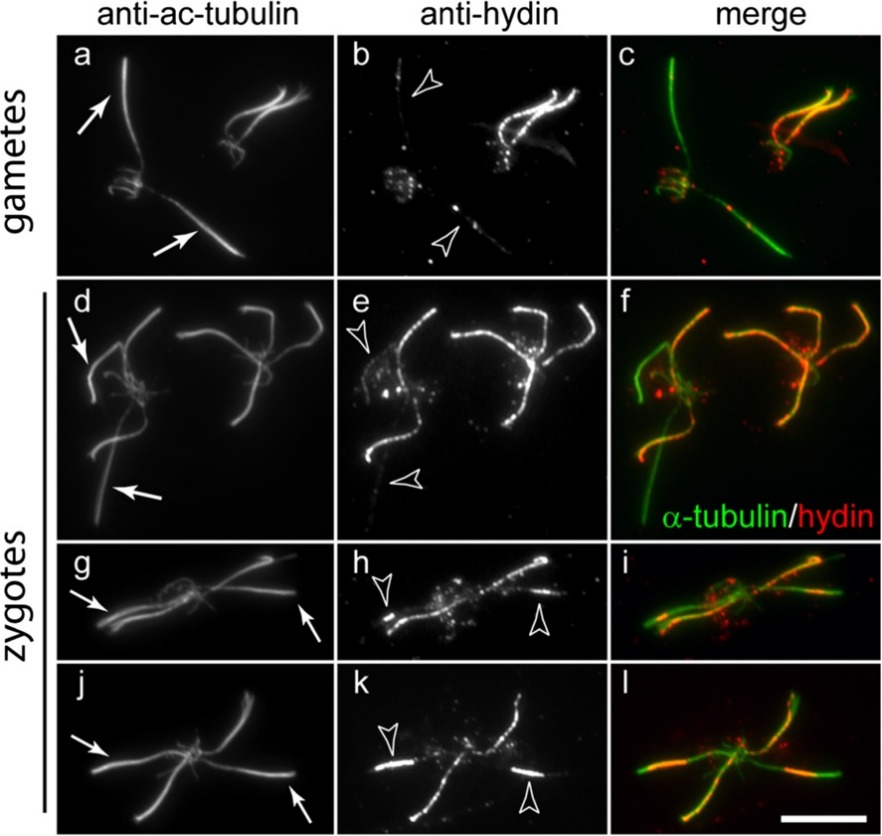
**Distribution of hydin during CP assembly.** Gametes **(a–c)** and zygotes **(d–l)** from a mating of the CP mutant *pf19* with *WT* (CC124) were analyzed by immunofluorescence microscopy using anti-acetylated α-tubulin and anti-hydin, as indicated. Arrows mark flagella of *pf19* gametes **(a)** or flagella derived from *pf19* in quadriflagellated zygotes **(d, g, and j)**. Arrowheads in **b** and **e**: flagella largely lacking hydin, indicating the absence of a CP. Arrowheads in **h** and **k**: hydin accumulation in subdistal regions of flagella derived from the CP mutant. Note that the accumulation occurs symmetrically in the two flagella of a given zygote. Bar = 10 μm.

By immunofluorescence microscopy, flagella of *pf19* gametes contained only a few small foci of hydin and thus could be easily distinguished from those of *WT* gametes, which showed hydin staining almost along the entire length of the flagella (Figure 3a–c). During mating of *C. reinhardtii*, the time span between mixing of the gametes and the actual cell fusion event ranges from a few seconds to many minutes. Therefore, zygotes of different ages are observed in the same sample. Figure 3d–f shows an early *pf19 x WT* zygote with two hydin-positive flagella provided by the *WT* parent and two hydin-deficient flagella derived from the *pf19* parent. Next to it is a late zygote which possesses hydin in all four flagella, indicating that CPs have been formed in the flagella formerly lacking CPs. Zygotes with hydin in all four flagella were not observed in samples fixed at 10 min but were abundant in samples fixed 60 min after mixing of the gametes. This suggests that hydin accumulation in the mutant-derived flagella progresses with time, allowing us to assess the age of zygotes based on the length of the region into which hydin has been incorporated. Surprisingly, hydin staining was restricted to middle or subdistal regions of the formerly mutant flagella at intermediate ages (Figure 3h,k); usually, the accumulation occurred symmetrically in the two flagella of a given zygote. Such short hydin signals were generally more intense when compared to those of the *WT* flagella of the same quadriflagellate. This may be because proteins associated with hydin are reduced or absent during early stages of CP assembly, thus increasing accessibility of the antibodies to hydin.

### The CP assembles subdistally during the repair of CP-deficient flagella

**Figure 4:**
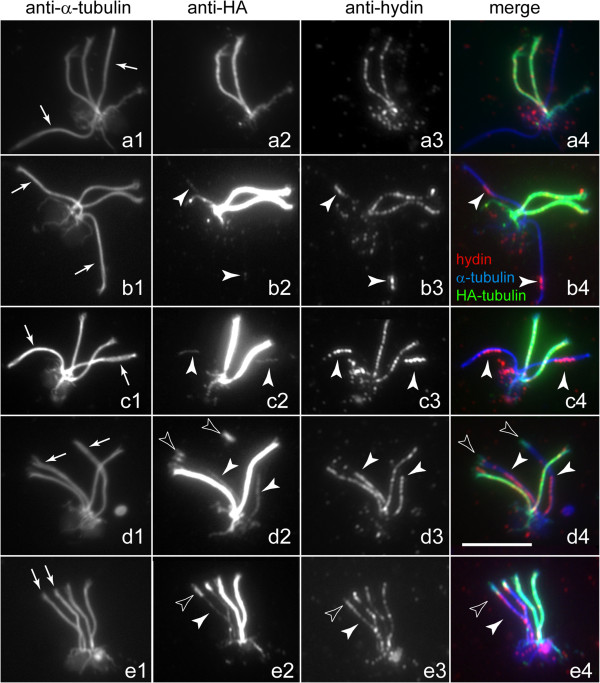
**Microtubule formation during CP assembly.** Zygotes obtained by mating *pf15* with a *WT* strain expressing triple HA-tagged α-tubulin were analyzed by immunofluorescence microscopy using anti-α-tubulin **(a1–e1)**, anti-HA **(a2–e2)**, and anti-hydin **(a3–e3)**. Merged images are shown in **a4–e4**. Arrows in **a1–e1**: flagella derived from *pf15*. Filled arrowheads: developing CP as detected with anti-HA and anti-hydin. Open arrowheads: incorporation of HA-tubulin at the distal end of flagella derived from *pf15*. Bar = 10 μm.

Our observation that hydin appeared subdistally to the tips during repair of formerly CP-deficient *pf19* flagella raised the question whether the distribution of hydin truly reflects the location of the CP microtubules or whether these microtubules are actually longer and only partially decorated with hydin. To address this, we carried out mating experiments using a *WT* strain expressing HA-tagged α-tubulin [40], which enabled us to image the assembly of CP microtubules together with hydin. After cell fusion, HA-tubulin present in the shared cytoplasm of the zygote is available for transport and incorporation into the flagella derived from the non-HA acceptor strain. Following mating of the CP-deficient mutant *pf15* to the α-tubulin-HA donor strain, the resulting quadriflagellated zygotes were analyzed by triple immunofluorescence using anti-α-tubulin, anti-HA, and anti-hydin (Figure 4). It has been previously shown that the epitope-tagged tubulin slowly incorporates into the tip regions of the outer doublet and CP microtubules of *WT* flagella; this process is due to turnover at the tips of steady-state flagella [40,41]. Thus, the age of zygotes can be assessed based on the length of the region into which HA-tubulin has been incorporated into the acceptor flagella. Early zygotes possessed two *WT* flagella (HA positive and hydin positive) and two CP-deficient flagella (HA negative and hydin negative; Figure 4a). HA-tagged tubulin first became apparent in a subdistal region of the formerly mutant flagella, indicating the formation of new microtubules (arrowheads in Figure 4b,c). These thread-like signals co-localized with hydin over their entire length. We conclude that these structures represent the developing CP and that hydin is added early to the developing CP. In older zygotes, as indicated by the presence of HA-tagged tubulin in the tip region of the formerly mutant flagella, the hydin and HA-tubulin signals were longer, indicative of CP elongation (Figure 4d). At this time, the developing CP was observed in various positions along the length of the flagella with a preference for the proximal region (Figure 4d). This variability may indicate that the developing CP slides inside the axonemal cylinder, possibly driven by the onset of flagellar bending. In even later stages, the distribution of HA-tubulin into the formerly mutant flagella resembled a drumstick consisting of the thin CP and a broader distal segment representing incorporation of HA-tubulin into the outer doublet microtubules (Figure 4e). The developing CPs were mostly of similar length and in similar positions in the two formerly mutant flagella of a given zygote (Figure 3h,k and Figure 4b,c), suggesting a spatiotemporal coordination of CP assembly.

**Figure 5:**
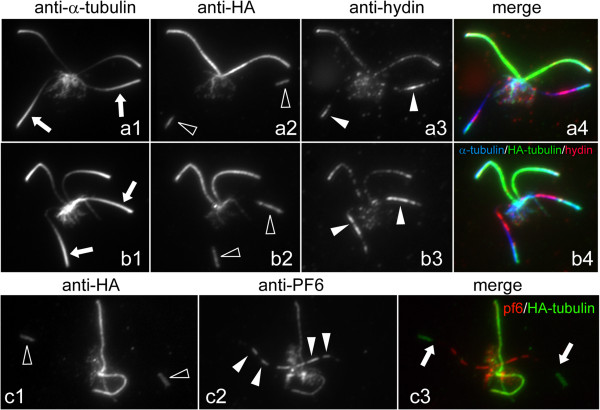
**Localization of hydin in *pf18 *x wild type dikaryons.** Zygotes from a mating of *pf18* with a *WT* strain expressing α-tubulin fused to a triple HA-tag were analyzed by immunofluorescence microscopy using anti-α-tubulin **(a1, b1)**, anti-HA **(a2, b2)**, and anti-hydin **(a3, b3)** or anti-HA **(c1)** and anti-PF6 **(c2)**. Merged images are shown in **a4**, **b4**, and **c3**. Arrows: flagella derived from *pf18*. Open arrowheads: incorporation of HA-tubulin at the distal end of flagella derived from *pf18*. Filled arrowheads: hydin **(a3, b3)** or PF6 **(c2)** in flagella derived from *pf18*. CP assembly is not apparent in flagella derived from *pf18*. Bar = 5 μm.

### Hydin is transported into CP-deficient flagella in the absence of detectable CP formation

In the mutant *pf18*, which is still uncharacterized at the molecular level, the CP also is replaced by an amorphous central core. However, in contrast to the situation with *pf15* and *pf19*, motility is not restored to *pf18* flagella following mating to *WT*[25], indicating that a functional CP fails to assemble in these flagella in the zygote. To determine if hydin is transported into *pf18* flagella even in the absence of CP formation, we mated *pf18* with *WT* cells expressing HA-α-tubulin and analyzed the zygotes by triple immunofluorescence microscopy (Figure 5). Even in late *pf18* x *WT* zygotes, as indicated by considerable incorporation of HA-tubulin into the distal portions of the formerly mutant flagella, the thread-like HA-positive structures observed during rescue of *pf15* flagella were absent (Figure 5a2,b2). This is in agreement with the reported lack of motility of *pf18*-derived flagella in *pf18* x *WT* zygotes and confirms that the thread-like HA-tubulin structures observed during the repair of *pf15* flagella represent the CP microtubules. Importantly, hydin, which is largely absent from the flagella of *pf18* gametes (not shown), accumulated in subdistal and middle regions of the formerly *pf18* flagella, where the protein appeared to be more dispersed than during repair of *pf15* flagella (Figure 5a3,b3). Apparently, fusion of the gametes activates transport of hydin, presumably by IFT, to the tip of the *pf18* flagella in anticipation of CP assembly, which never occurs for reasons not yet understood. Because no CP microtubules are present, hydin cannot be accumulating in this region simply by virtue of binding to a newly formed CP microtubule.

**Figure 6:**
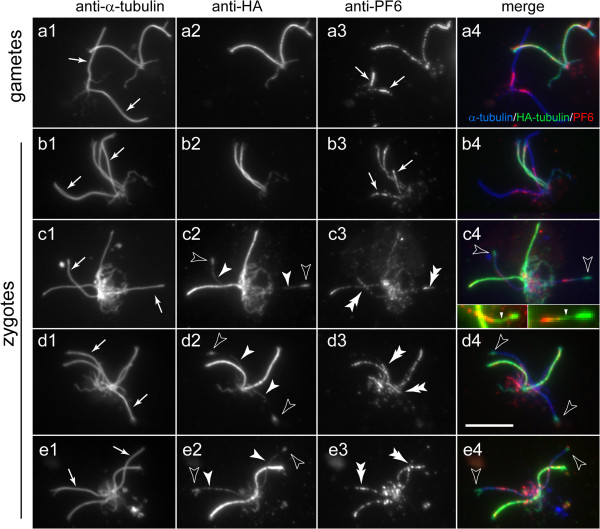
**Distribution of PF6 during *de novo *CP assembly.** Gametes and zygotes from a mating of *pf15* cells with *WT* cells expressing triple HA-tagged α-tubulin were analyzed by immunofluorescence microscopy using anti-α-tubulin **(a1–e1)**, anti-HA **(a2–e2)**, and anti-PF6 **(a3–e3)**. Merged images are shown in **a4–e4**. Arrows in **a1–e1**: flagella of *pf15* gametes **(a1)** or zygotic flagella derived from *pf15***(b1–e1)**. Filled arrowheads in **c2–e2**: developing CP as detected with anti-HA. Open arrowheads in **c–e**: incorporation of HA-tubulin at the distal ends of flagella derived from *pf15*. Arrows in **a3** and **b3**: PF6 is present in the proximal regions of flagella of *pf15* gametes and of flagella derived from *pf15* in early quadriflagellates. Double arrowheads: position of PF6 during CP development. Inserts in **c4**: PF6 (red) is only present in the proximal regions of the assembling CP; accumulation of HA-tubulin (green) at the distal tip represents tubulin turnover in the outer doublets. Arrowheads: PF6-deficient regions of the newly formed CP. Bar = 10 μm.

### PF6 trails hydin during CP assembly

To determine if our observations on hydin during *de novo* CP assembly are representative for other CP proteins, we analyzed the distribution of PF6, a CP-specific protein associated with a projection (C1a) of the C1 microtubule [28,42], in flagella of zygotes resulting from a cross between *pf15* gametes x *WT* gametes expressing HA-tubulin (Figure 6). In contrast to hydin, PF6 was present in most *pf15* gametic flagella, where it was concentrated predominately in the proximal region (Figure 6a). In early zygotes, the PF6 signal in the flagella derived from *pf15* was less dense and extended more distally than in the gametic flagella, probably indicative of a redistribution of the PF6 already present in the flagella (Figure 6b). The PF6 signal overlapped with the proximal regions of the developing CPs as visualized by HA-tubulin staining (Figure 6c). In later stage zygotes, PF6 was largely restricted to the proximal portions of elongated and even full-length CPs (Figure 6d,e). The data suggest that in *pf15* x *WT* zygotes, i) CP repair draws at least in part on a pool of PF6 already present in the flagella; ii) the addition of hydin to the CP precedes that of PF6; and iii) PF6 is added to the developing CP in a base-to-tip fashion. Notably, a similar redistribution of PF6 was also observed in the mutant-derived flagella of *pf18* x *WT* zygotes in the absence of apparent CP formation (Figure 5c). Thus, both hydin and PF6 redistribute in *pf18*-derived flagella without the formation of CP microtubules as indicated by the absence of HA-tubulin incorporation.

### PF6 is added tip-to-base onto PF6-deficient CPs

**Figure 7:**
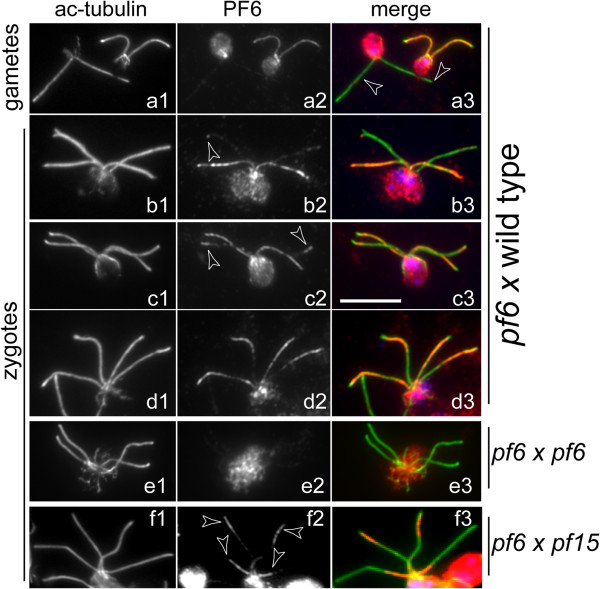
**Distribution of PF6 during repair of *pf6 *mutant flagella.** Gametes **(a)** and zygotes **(b–f)** from matings of *pf6* with *WT***(a–d)**, *pf6* with *pf15***(f)**, and, as a control for antibody specificity, *pf6* with *pf6***(e)** were labeled with antibodies to acetylated α-tubulin and PF6. Merged images, mostly counterstained with DAPI to visualize the nuclei, are shown in the third column. Arrowheads in **b2** and **c2**: incorporation of PF6 near the tip of zygotic flagella derived from *pf6*. In **f**, note the strikingly different distribution of PF6 in the lower pair of flagella derived from the CP-deficient strain *pf15* vs. the upper pair of flagella derived from the PF6-deficient strain. Bar = 10 μm.

Cells of the mutant *pf6* have flagella with CPs but the C1 microtubule of the CP lacks the C1a projection, which includes PF6 and several other proteins [28,42,43]. To test whether PF6 assembly generally progresses from base to tip, we mated *pf6-1* gametes to *WT* cells; this allowed us to determine how PF6 is added to an existing CP that initially lacks PF6. In *pf6 x WT* zygotes, PF6 was first detected at the tip of the formerly mutant flagella (Figure 7b,c). In most zygotes, the intensity of the PF6 signal in the *pf6*-derived flagella decreased toward the flagellar base (Figure 7d). Therefore, PF6 is added gradually in a tip-to-base fashion to PF6-deficient CPs. The data suggest that PF6 is first transported to the flagellar tip, presumably by IFT, and then moves proximally inside the axonemal lumen to its CP docking site. To verify the specificity of the anti-PF6 antibody, we mated *pf6* to itself; as expected, PF6 was absent from all four flagella of the resulting zygotes (Figure 7e). To confirm the differences in the distribution of PF6 during repair vs. *de novo* assembly of the CP, we mated *pf15* (PF6 present, no CP) to *pf6* (no PF6, CP present). In a given *pf6 x pf15* zygote, PF6 was strongly localized to the tip region of two flagella and to the basal region in the other two flagella (Figure 7f).

### Proteins associated with the C1 microtubule are present in CP-deficient flagella

**Figure 8:**
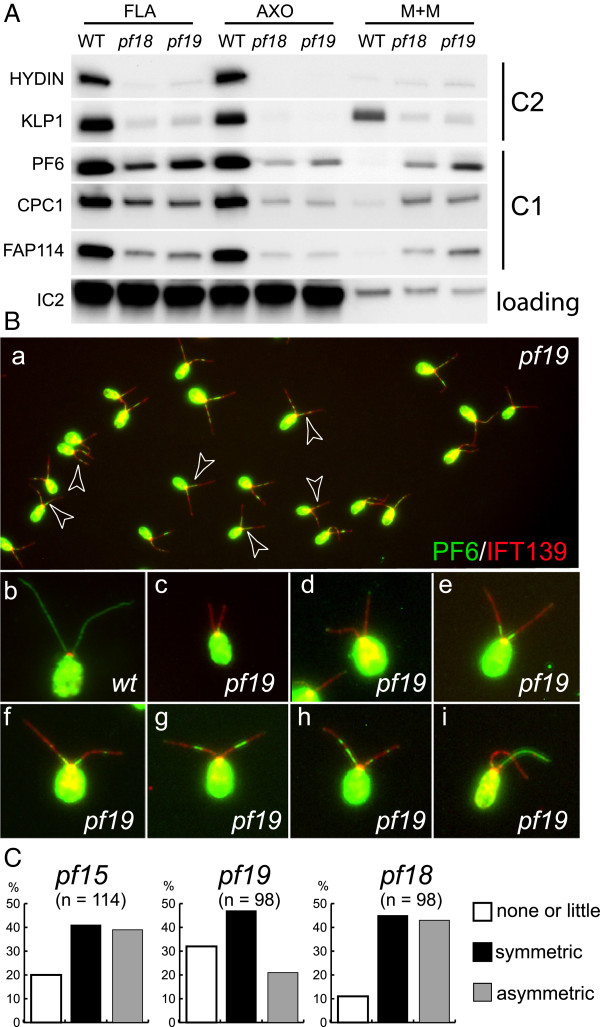
**C1-associated proteins are present in CP-deficient flagella. (A)** Western blot of flagella (FLA), axonemes (AXO), and the membrane + matrix fractions (M + M) isolated from *WT* and the CP-deficient mutants *pf18* and *pf19* probed with antibodies as indicated. Only traces of the C2 proteins hydin and KLP1 were detected in *pf18* and *pf19* flagella and both proteins were almost completely released into the detergent extract (M + M). In contrast, significant amounts of the C1 proteins PF6, CPC1, and FAP114 were present in the CP-deficient flagella and a significant proportion of these proteins remained in the axonemal fraction following detergent extraction. The outer dynein arm intermediate chain IC2 was used as a loading control. **(B)** To analyze the distribution of PF6 in CP-deficient flagella, methanol-fixed vegetative *WT***(b)** and *pf19* cells **(a, c–i)** were labeled with antibodies to PF6 and IFT139. Arrowheads (subpanel **a**) mark cells with a nearly symmetrical distribution of PF6 in both flagella. Note the accumulation of IFT139 in *pf19* flagella compared to *WT* flagella. **(C)** The distribution of PF6 in *pf15*, *pf18*, and *pf19* flagella was scored as reduced or absent **(**e.g. **B c, d)**, nearly symmetrical **(**e.g. **B e, f, g)**, or asymmetric **(**e.g. **B h, i)** within the two flagella of a given cell.

The above immunofluorescence microscopy results showed that some PF6, a C1-associated protein, was present in *pf15* gametic flagella, whereas hydin, a C2-associated protein, was almost completely absent. To confirm and extend these observations, we probed western blots of isolated flagella from *WT*, *pf15* (not shown), *pf18*, and *pf19* vegetative cells with antibodies to these and other CP proteins (Figure 8A). The blots revealed that the amounts of PF6 present in CP-deficient flagella corresponded roughly to 20% to 25% of that present in *WT* flagella. Similarly, CPC1, the major structural protein of the C1b projection [34], and FAP114, which is part of the C1a projection [28], were found in CP-deficient flagella in amounts roughly corresponding to one quarter of that in *WT*. In agreement with our fluorescence-based microscopic analysis of gametic flagella, only traces of hydin were detected in the isolated flagella from CP-deficient vegetative cells. Similarly, only small amounts of KLP1, a kinesin-like protein associated with the C2 microtubule [35], were present in the CP-deficient flagella. In summary, these western blots indicated that three proteins associated with the C1 microtubule are present in CP-deficient flagella, albeit in amounts which would be insufficient to assemble a full-length CP. In contrast, the C2-associated proteins hydin and KLP1 are largely absent from these flagella. The results indicate that C1 proteins are specifically transported into or retained in the CP-deficient flagella.

We also investigated if the CP proteins present in the CP-deficient flagella were released when the flagellar membrane was removed by treatment with non-ionic detergent. The small amounts of the C2 proteins hydin and KLP1 present in these flagella were almost completely released into the detergent-soluble membrane + matrix fraction (Figure 8A). In contrast, only slightly more than half of the C1 proteins PF6, CPC1, and FAP114 were released. These results suggest that a significant proportion of the C1 proteins are trapped within the axoneme, probably in the central core, which also is not released by detergent treatment [23]. The C2 proteins may be associated with moving IFT particles, nearly all of which are released from *WT* flagella under these conditions.

Finally, using immunofluorescence microscopy, we confirmed the presence of PF6 in the flagella of vegetative *pf15*, *pf18*, and *pf19* cells (Figure 8B,C). Interestingly, the distribution of PF6 in the two flagella of a given cell was often quasi symmetric. This pattern could arise if PF6 is transported into the growing flagella at a specific time and then becomes concentrated in a particular region of the central core, wedged between proteins transported in earlier (located more proximally) and later (located more distally). This idea could not be tested here because available antibodies to other C1 proteins are not suitable for immunofluorescence microscopy.

### IFT proteins accumulate in CP-deficient flagella

**Figure 9:**
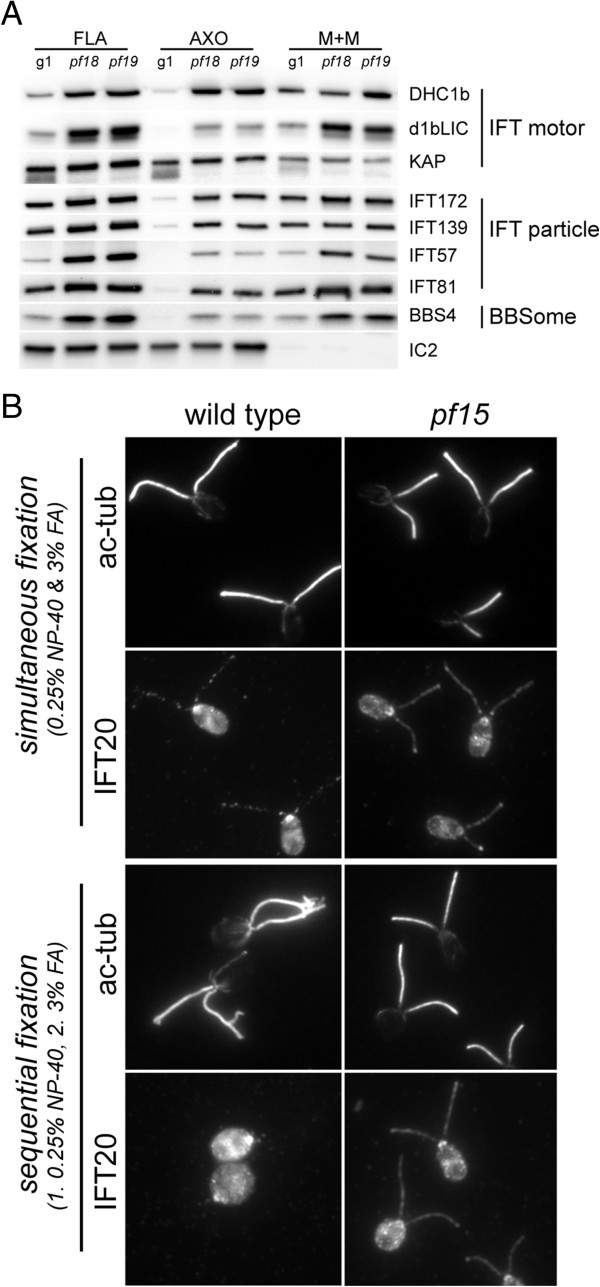
**IFT proteins accumulate in CP-deficient flagella. (A)** Western blot probing isolated flagella (FLA), axonemes (AXO), and the membrane + matrix fraction (M + M) of *WT* (g1) and the CP-deficient mutants *pf18* and *pf19* with the antibodies indicated. Similar results were obtained for *pf15* (not shown). **(B)** Immunofluorescence microscopy of *WT* and *pf15* cells extracted with detergent and fixed with formaldehyde either simultaneously (top) or sequentially (bottom) and then stained with antibodies to acetylated tubulin and IFT20. Note retention of IFT20 in *pf15* but not *WT* axonemes extracted before fixation.

In the course of the above studies, we noticed that proteins of IFT particle complex A (IFT139), IFT complex B (IFT57, IFT81, and IFT172), the IFT retrograde motor (DHC1b and D1bLIC), and the BBSome (BBS4), an IFT adaptor [30,44], were significantly enriched in CP-deficient flagella of *pf15*, *pf18*, and *pf19* (Figure 9A; *pf15* data are not shown). KAP, a component of the anterograde IFT motor [45], was present in near normal or slightly elevated amounts in *pf18* and *pf19* flagella. IFT proteins are almost completely extracted from *WT* flagella by detergent treatment (Figure 9A). In contrast, substantial amounts of the IFT proteins present in the CP-deficient flagella remained with the axonemes even after prolonged detergent treatment (30 min on ice). Immunofluorescence microscopy confirmed that IFT20 was rapidly extracted from detergent-treated *WT* flagella, whereas it remained attached over the entire length of detergent-treated CP-deficient flagella (Figure 9B); similar results were obtained for IFT172, IFT81, IFT57, and D1bLIC (not shown). These results indicate that CP-deficient flagella accumulate IFT proteins in a pool that appears to be trapped within the axoneme.

### The lumens of CP-deficient flagella contain IFT proteins that are removed during CP assembly

**Figure 10:**
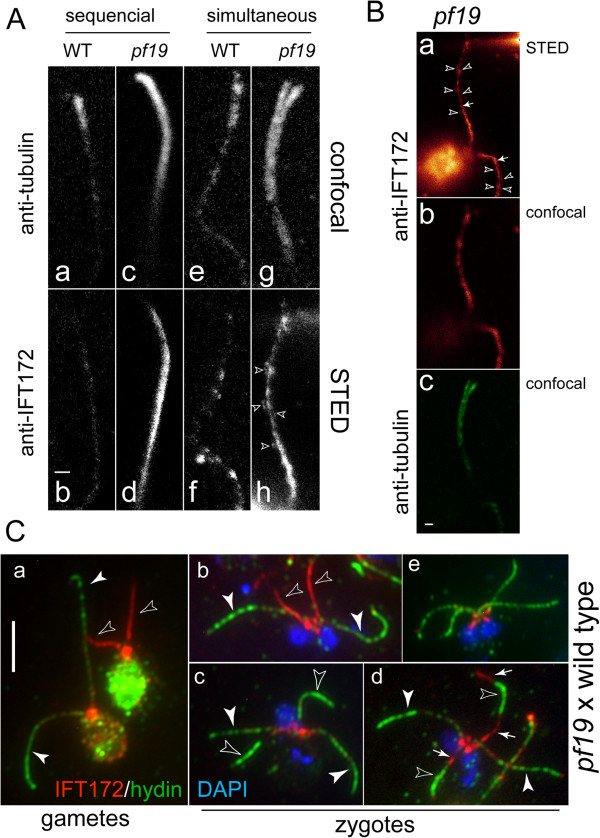
**IFT172 is part of the fibrous core of *pf19 *flagella. (A)** Confocal **(a, c, e, g)** and STED **(b, d, f, h)** images of *WT* flagella stained with anti-β-tubulin **(a, c, e, g)** and anti-IFT172 **(b, d, f, h)**. Cells were extracted and fixed either sequentially **(a-d)** or simultaneously **(e-h)**. Arrowheads in **h**: IFT172-containing particles flanking a central rod containing IFT172; note the absence of such particles in the flagella (**b** and **d**) derived from cells extracted first with detergent. Note also that the central IFT172-containing core, present in *pf19* flagella **(d)** but not *WT* flagella **(b)**, persists after detergent extraction **(h)** and is absent from the distal end of the cilium (compare **c** and **d**). **(B)** Overview of the cell corresponding to **g**/**h** showing the IFT172 signal in STED **(a)** and confocal illumination **(b)** and the tubulin signal in confocal illumination **(c)**. Arrowheads: IFT172-containing particles flanking the central rods (arrows), which are stained strongly by the IFT172 antibody. **(C)***WT* and *pf19* gametes **(a)** and the resulting zygotes **(b–e)** were stained with antibodies to hydin (green) and IFT172 (red); DAPI staining is shown in blue. Open arrowheads: flagella of *pf19* gametes **(a)** or zygotic flagella derived from *pf19***(b–d)**. Closed arrowheads: flagella of *WT* gametes **(a)** or zygotic flagella derived from *WT***(b–d)**. Small arrows in **d**: residual IFT172 flanking the developing CP as visualized by anti-hydin. Bar = 5 μm.

To determine where in the mutant flagella the accumulated IFT proteins are located, we used STED microscopy to image IFT complex B protein IFT172 in *WT* and *pf19* flagella at high resolution (Figure 10A,B). When cells were extracted with detergent prior to fixation, IFT172 was largely removed from *WT* flagella whereas a strong, continuous, rod-like signal was observed in *pf19* flagella (Figure 10Ab,d). When cells were simultaneously permeabilized and fixed, a punctate staining was observed along *WT* flagella, which we interpret as representing IFT particles on the outside of the axoneme (Figure 10Af). In *pf19* cells that were simultaneously permeabilized and fixed, the rod-like IFT172 signal extending nearly the length of the flagella was flanked by smaller spots; these details in the distribution of IFT172 were not resolved by standard confocal microscopy (Figure 10Ah,B). The spots are likely to represent IFT particles on the outside of the axonemal cylinder while the continuous rod-like signal indicates the presence of IFT172 in the center of the axoneme, probably in the fibrous core, the composition of which had not previously been defined.

If the insoluble IFT proteins are indeed part of the fibrous core that fills the lumen of CP-deficient flagella, the proteins should be removed from CP-deficient flagella during *de novo* CP assembly. To test this, we used immunofluorescence microscopy to examine the flagella of gametes and zygotes that were extracted with detergent prior to fixation. As shown above, such extraction removes the IFT particles that are located between the outer doublets and flagellar membrane of *WT* cells, but does not remove the fibrous core of CP-deficient flagella. As expected, IFT172 was largely absent from extracted flagella of *WT* cells but readily observed in extracted flagella of *pf19* gametes (Figure 10Ca). Early *pf19 x WT* zygotes had two flagella (derived from the *WT* parent) containing hydin and only traces of residual IFT172 and two flagella (derived from the *pf19* parent) largely devoid of hydin and containing IFT172 (Figure 10Cb). Flagella of older zygotes lacked detergent-insoluble IFT172 and had hydin in all four flagella (Figure 10Ce). Intermediate stages showed hydin concentrated in subdistal regions of the *pf19*-derived flagella; such flagella largely lacked IFT172 (Figure 10Cc). More rarely, residual IFT172 was observed flanking the regions of hydin accumulation (Figure 10Cd). Therefore, IFT172 was removed from the formerly CP-deficient flagella during CP formation. We conclude that IFT proteins are part of the fibrous core and are removed prior to or concomitant with *de novo* CP assembly.

### CP-deficient flagella are shorter than wild-type flagella

**Figure 11:**
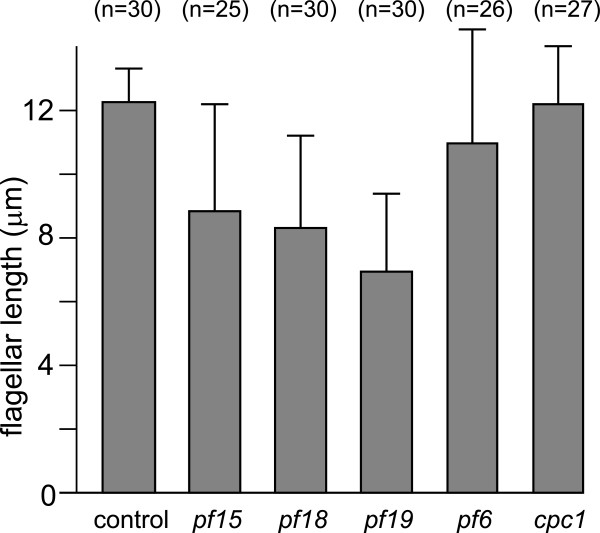
**Flagellar length is reduced in CP-deficient mutants.** Bar graph showing average steady-state flagellar length in *WT*, the CP-deficient mutants *pf15*, *pf18*, and *pf19*, and CP mutants *pf6* (lacking the C1a projection) and *cpc1* (lacking the C1b projection [34]). The number of vegetative cells scored is indicated. Error bars indicate the standard deviation.

IFT is required for the assembly and maintenance of flagella, raising the question of whether the accumulation of IFT proteins affected flagellar assembly by the CP-deficient mutant cells. In aerated cultures, vegetative *pf15*, *pf18*, and *pf19* cells showed a reduced flagellar length ranging from ~70% of *WT* length in *pf15* to less than 60% of *WT* length in *pf19* (Figures 8B, 9B, and 11). In cultures maintained on a rotary shaker, flagellar length was often reduced to less than 50% that of *WT* flagella. In contrast, the flagellar length of *pf6* cells or of the C1b projection mutant *cpc1* was not significantly different from that of *WT*. Thus, CP-deficient mutants have a modest short flagella phenotype. Interestingly, gametic flagella of the CP-deficient mutants are mostly of normal length and sometimes even exceed the length of *WT* flagella (Figure 4a1,b1); however, we noticed a tendency to form flagella only slowly or not at all in some gametes of the CP-deficient mutants (not shown).

### Two CPs with correct polarity can be formed in *pf14* flagella

**Figure 12:**
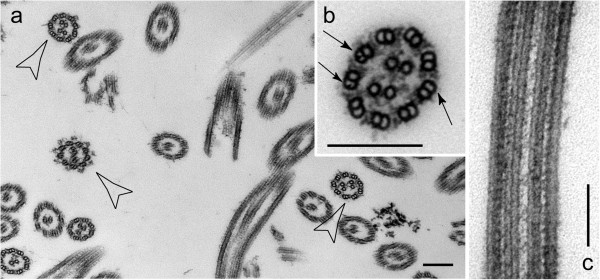
**Multiple CPs in pf14 flagella.** Standard transmission EM of isolated *pf14* axonemes. **(a)** Axonemes with four CP microtubules are marked. **(b, c)** Axonemes with two CPs in cross and longitudinal section. When two CPs are present, both always have correct, identical polarities, as seen in the cross section. Arrows point to beak-like projections in doublets 1, 5, and 6, indicating that the section is from the proximal ~1/3 of the flagellum [31].

Our observations on CP assembly raise questions about how the cells establish the correct number and polarity of CP microtubules. Previous studies have shown that in certain *C. reinhardtii* double and triple mutants in which extra space is available in the lumen of the axoneme, some cells will assemble more than a single CP [9]. To further explore the possibility that the CP can be formed without the requirement for a template, we examined isolated axonemes of the mutant *pf14*, which lacks radial spokes. Although not previously reported for this mutation in isolation, we observed numerous examples of axonemes with two CPs (Figure 12). Importantly, in every case (8 of 8 axonemes examined) both CPs had identical and correct polarities. The fact that both CPs are formed with correct polarity argues against the existence of a structurally defined CP-organizing center that templates a single CP in the correct location within the axoneme.

## Discussion

### The transport of CP proteins may determine the location of CP assembly

In regenerating flagella, the CP forms shortly after the start of outer doublet elongation, when the flagellum is less than ~0.8 μm long. Because CP assembly begins in such a tightly constrained space, we could not determine if assembly is initiated at a particular site – e.g., at the TZ or the flagellar tip. However, during repair of CP-deficient flagella in dikaryons formed by mating CP-deficient gametes to *WT* gametes, a fully functional CP is added to an existing full-length flagellum. In this case, CP formation commences in a subdistal region of the flagellum, indicating that CP assembly does not depend on proximity to the TZ or flagellar tip.

In both cases, it is likely that CP precursors are transported via IFT to the flagellar tip and then diffuse into the lumen of the axoneme, where they self-assemble into the CP. Indeed, we provide evidence that PF6, a subunit of the CP, is a cargo of IFT as indicated by its tip-to-base assembly onto existing PF6-deficient CPs. Such transport to the tip of the flagellum likely occurs for many axonemal precursors, as similar patterns have been observed for the assembly of the radial spoke protein RSP3 in radial spoke-deficient flagella of *pf14* x *WT* dikaryons [40] and for the assembly of DRC4-GFP, a component of the nexin-dynein regulatory complex, into flagella of the corresponding *pf2* mutant [46]. In very short regenerating flagella, the CP subunits would be deposited into the space where the future CP will form; in slightly longer flagella, they would be deposited near the site of CP elongation, assuming that the CP elongates by addition of new subunits to its distal tip. During repair of CP-deficient flagella, CP subunits will be similarly transported to the tip of the flagellum by IFT and enter the lumen of the axoneme, but here they will encounter the electron-dense core that extends nearly to the tip of the axonemes of such CP-deficient flagella. It is likely that this core impedes diffusion further down the lumen, so that the CP subunits self-assemble in the subdistal region of the flagellum. As the core is eventually replaced by the newly forming CP, it may be dissolved and some of its components incorporated into the new CP and others recycled for IFT.

### Control of CP number and polarity

Our finding that a CP initially forms in the subdistal region of the flagellum during repair of CP-deficient flagella indicates that the CP is capable of self-assembling without templating by a CP-organizing center at the base or tip of the flagellum. Such a CP-organizing center, if it existed, would presumably provide two nucleation sites onto which the two CP microtubules would polymerize. However, two CPs (containing four central microtubules with projections) have been observed in *C. reinhardtii pf14 pf6*, *pf14 cpc1*, and *pf14 pf6 cpc1* double and triple mutants, which lack the radial spokes (*pf14*) and one or two of the two major C1 projections [9]. Such mutants provide more space in the axonemal lumen combined with a reduced size of the CP. We report here that the lack of radial spokes in *pf14* alone is sufficient to accommodate four CP microtubules with attached projections and correct orientation in the axonemal lumen. Two CPs also have been observed in nodal cilia of the rabbit notochord [47], which apparently lack bona fide radial spokes. Flagella of *sas-6* mutants (*C. reinhardtii bld12*) sometimes have axonemes consisting of 10 doublets and two CPs whereas CPs are generally absent from flagella with 8 doublets of the same mutant [9,48]. This variability of CP microtubule number argues against the presence of a CP-organizing center that nucleates precisely two microtubules. It rather indicates that the space available inside of the axonemal cylinder plays a role in the control of CP number.

CP microtubules have the same polarity as the outer doublet microtubules [14], and one can imagine various mechanisms to establish this polarity. At the beginning of flagellar growth, the TZ could capture one end of the nascent CP and thereby ensure its correct orientation. However, such a mechanism is unlikely during the repair of CP-deficient flagella when CP assembly is spatially separated from the TZ. The radial spokes are in contact with the CP apparatus and in theory could function in positioning the CP microtubules with respect to axonemal polarity. CP orientation, however, is correct in radial spoke-deficient mutants (Figure 12), indicating that radial spokes are expendable for this process. The CP is linked via a special cap structure to the membrane covering the flagellar tip, but this association forms late during CP assembly and thus is unlikely to determine CP polarity. Finally, CP proteins are likely to be released from IFT at the ciliary tip regardless of whether flagella are short and growing or full length. This could result in a tip-to-base gradient of CP precursors in the axonemal lumen, which hypothetically could force the CP apparatus to form in the correct orientation.

### Differences between *de novo* assembly and repair of CPs and between C1 and C2 subunit accumulation in the flagellar core

Two additional observations provide potentially important information relevant to the process of CP protein transport and CP repair. First, we observed that the C1 protein PF6 is added progressively tip-to-base during repair of a CP lacking only the C1a projection but is assembled base-to-tip onto the newly forming microtubules during *de novo* CP assembly. This difference almost certainly is due to the different distributions of PF6 in the two cases. In the first case, there is no PF6 accumulated in the flagella, and new PF6 must be provided by IFT, which delivers it to the tip of the flagellum. In the second case, there is often a substantial pool of PF6 accumulated in the proximal part of the flagellum, apparently in the central core, and this pool may be drawn upon as CP assembly proceeds and the core is dissolved, thus providing PF6 subunits from a proximal source within the axonemal lumen.

Second, we found that all three C1 proteins examined were accumulated in the central core of CP-deficient flagella, while neither of the C2 proteins examined was accumulated. This may reflect a stronger affinity of C1 microtubule subunits for each other and/or for IFT particles, leading to their aggregation into the central core; indeed, the C1 microtubule is more stable than the C2 microtubule when axonemes are extracted with the anionic detergent Sarkosyl [49]. Alternatively, the difference may reflect a fundamental difference in the way the C1 and C2 microtubules are assembled.

### IFT proteins occupy the fibrous core of CP-deficient flagella

IFT particle and motor proteins are enriched in CP-deficient flagella. Super-resolution microscopy, dikaryon rescue experiments, and biochemical analyses indicate that at least a portion of these IFT proteins are contained within the lumen of the CP-deficient flagella. This lumen has a fibrous core [23,24], and we propose that this material consists in large part of IFT proteins. The absence of the CP might allow IFT particles to enter the lumen of the axoneme at the tip of the flagellum; if exit through the TZ and basal body were restricted, the particles would then accumulate inside the axonemal cylinder. Electron-opaque material which might be similar to the fibrous core observed in *C. reinhardtii* is visible in CP-deficient nodal cilia, primary cilia such as the connecting cilium, and mutant cilia in various organisms [16,50-52]. Thus, the presence of a central core possibly containing IFT proteins and CP precursors may be a general feature of cilia lacking a CP.

The anomalous distribution of IFT proteins in *C. reinhardtii* CP-deficient mutants puts a cautionary note on using these mutants for the *in vivo* analysis of IFT. Indeed, vegetative cells of *pf15, pf18,* and *pf19* often assemble shorter-than-normal flagella that could be due to a defect in IFT. In a simple model, IFT particles could get trapped in the void left vacated by the CP as flagella elongate, and this redistribution of IFT particles could affect steady-state flagellar length – e.g., by reducing the pool of IFT particles available to build and maintain the flagellum [53]. Alternatively, major ultrastructural defects in general or impaired flagellar motility might result in an accumulation of IFT proteins in flagella through induction of a compensating repair process. Rompolas et al. reported that the *C. reinhardtii* homologue of the lissencephaly protein LIS1 accumulates in flagella of motility mutants as well as in wild-type flagella under high viscous load where flagellar motility is reduced [54]. They suggested that cells sense the absence of or changes in flagellar motility and respond by moving LIS1 into the flagella to support the activity of axonemal dynein, the LIS1 binding partner under high-load conditions. The data raise the intriguing possibility that IFT monitors the function and structural integrity of flagella. It will be of interest to determine if other classes of structural mutants, e.g., radial spoke or inner and outer dynein arm mutants, also accumulate IFT proteins, and if so, if there is a correlation with the degree of accumulation and the length of their flagella.

### CP self-assembly may explain the phenotype of katanin-defective mutants

Our finding that CP formation apparently occurs via self-assembly, without templating, provides a possible explanation for why katanin defects affect formation of the CP but not the outer doublets in *pf15* and *pf19*. The minus ends of the outer doublet microtubules are stabilized by the basal body, whereas the CP microtubules lack an ultrastructurally discernible cap at their minus ends. If the CP minus end is dynamic, allowing the loss of tubulin subunits, the critical concentration of tubulin for the formation and elongation of the CP microtubules will be higher than that for elongation of the outer doublets. Epitope-tagged katanin p80 localizes to the basal bodies, where katanin’s microtubule-severing activity could act on cytoplasmic microtubules organized around the basal bodies to generate unprotected, depolymerizing microtubule minus ends, thus increasing the concentration of tubulin dimers in the locale where IFT cargo loading occurs [55]. This locally increased tubulin concentration could result in an increased concentration of free tubulin inside the flagellum [56], bringing it to the higher levels necessary to support the nucleation and assembly of the CP microtubules. In the absence of katanin, intraflagellar free tubulin levels may be high enough to support outer doublet elongation but not CP assembly. Katanin subunits also have been shown to be present in cilia and flagella, apparently attached to the outer doublets [21,57], and it has been proposed that katanin could provide tubulin for CP assembly by trimming the distal ends of the outer doublet microtubules [57]. This could increase the concentration of tubulin near the ciliary or flagellar tip, promoting CP nucleation in the distal region of the organelle. However, in our dikaryon rescue experiments, HA-tagged tubulin provided by the *WT* parent was incorporated into the developing CP well before the epitope-tagged tubulin became apparent in the outer doublet microtubules. This suggests that the tubulin used for CP assembly is imported directly from the cell body without assembling first onto the outer doublet microtubules.

## Conclusions

The CP apparatus is required for the regulation of axonemal dyneins and is critical for the motility of 9 + 2 cilia and flagella. The basal body templates the nine outer doublet microtubules, but how the assembly of the CP microtubules is initiated has been unclear. To analyze CP assembly independently of outer doublet assembly, *C. reinhardtii* CP-deficient mutants *pf15* (defective in katanin p80) and *pf19* (defective in katanin p60) were mated to *WT* cells. In the resulting quadriflagellate zygotes, CP assembly was first apparent in subdistal regions of the formerly mutant flagella. We conclude that the CP self-assembles without requiring a template at either the transition zone or the flagellar tip. Proteins of the IFT machinery accumulate in the lumens of the axonemes of CP-deficient mutants; this imbalance in the distribution of IFT proteins may contribute to the reduced length observed for flagella of CP-deficient vegetative cells.

## Abbreviations

CP: Central pair; HA: Hemagglutinin; IFT: Intraflagellar transport; STED: Stimulated emission depletion microscopy; TEM: Transmission electron microscopy; TZ: Transition zone; WT: Wild type.

## Competing interests

The authors declare that they have no competing interests.

## Authors’ contributions

KFL designed and carried out the experiments. TJG assisted with the STED microscopy. KFL and GBW interpreted the results and wrote the manuscript. All authors read and approved the final manuscript.
